# Incarceration of the gravid uterus: a case report and literature review

**DOI:** 10.1186/s12884-019-2549-3

**Published:** 2019-11-08

**Authors:** Cha Han, Chen Wang, Lulu Han, Guoyan Liu, Huiyang Li, Fuman She, Fengxia Xue, Yingmei Wang

**Affiliations:** Department of Gynecology and Obstetrics, Tianjin Medical University General Hospital, No. 154 Anshan Road, Heping District, Tianjin, People’s Republic of China 300052

**Keywords:** Retroverted uterus, Pregnancy, Incarceration, Pelvic adhesions, Complications

## Abstract

**Background:**

Incarceration of the gravid uterus is a rare obstetric disorder that contributes to pregnancy-related complications. To understand its clinical characteristics and managements, we have reviewed the etiology, risk factors, clinical characteristics and current treatments of an incarcerated gravid uterus based on 162 cases reported in the English language literature, including our patient.

**Case presentation:**

A 25-year-old primigravida, with a history of lymphatic tuberculosis, infertility due to blocked fallopian tubes and received in vitro fertilization. The patient presented with urine retention and lower abdominal pain in the early second trimester. Uterine incarceration was diagnosed based on pelvic examination and abdominal ultrasound. A Foley catheter was placed and manual reposition was successful. No episode of retention was experienced after the further enlargement of the uterus and its ascent. A healthy infant was delivered vaginally on 38th week of pregnancy.

**Conclusions:**

Uterine incarceration due to pelvic adhesions is rare and, because of it non-specific clinical presentations, is often misdiagnosed. Abdominal ultrasound is instrumental for the diagnosis because it can directly image the disturbed uterine and pelvic anatomy. There are limited treatment options for uterine incarceration, but definitive diagnosis allows procedures to treat and to reduce severe complications of uterine incarceration.

## Background

Uterine retroversion is recognized as a normal variant and its prevalence is reported to be up to 15% of pregnancies in the first trimester [1]. In most cases, retroversion can spontaneously return to a normal axial position by 14th week of gestation when the gravid uterus grows into the abdominal cavity. In rare cases, the uterus remains retroverted and becomes retroflexed between the subpromontory sacrum and pubis in the pelvic cavity, potentially caused by uterine anomalies, fibroids, pelvic adhesions or a deep sacral cavity with a prominent promontory. Failures to timely diagnose and properly treat uterine incarceration often result in obstetric complications. Here we report a case of incarceration of a retroverted uterus with a history of in vitro fertilization and embryo transfer (IVF-ET). We also reviewed previous reports in the literature to highlight the importance for early recognition and prompt managements in improving outcomes for pregnant women with the condition, especially those who have difficulty becoming pregnant with assisted reproductive technology (ART).

## Case presentation

A 25-year-old patient, gravida 1, para 0, first presented to the regional hospital at 16 weeks of gestation and with chief complaints of vaginal bloody discharge for 6 days, unable to urinate, and mild lower abdominal pain. The patient was diagnosed with lymphatic tuberculosis at 17 years of age, but had no history of sexually transmitted diseases, pelvic inflammatory diseases, endometriosis, uterine leiomyomas, deep sacral concavity, surgery, or congenital uterine malformations, such as uterus didelphys. The patient experienced infertility for two years caused by bilateral obstruction of the fallopian tubes with uterine retroversion, which was detected by hystero-salpingo-graphy in the regional hospital. She eventually became pregnant by follicular aspiration and IVF-ET, and had been treated with daily intramuscular progesterone since the procedure. Subsequent ultrasound scans revealed normal pregnancy progression.

At 15 weeks and 2 days of gestation, small amounts of bloody vaginal discharge persisted for one day, and an ultrasound showed that the placenta covered the internal os of the cervix. She was then admitted with a diagnosis of a low position of the placenta and continued receiving progesterone therapy. She experienced worsening lower abdominal pain and difficulty with urination on fifth day after admission and was transferred to our tertiary hospital at gestational age 16 weeks and 1 day.

**Fig. 1:**
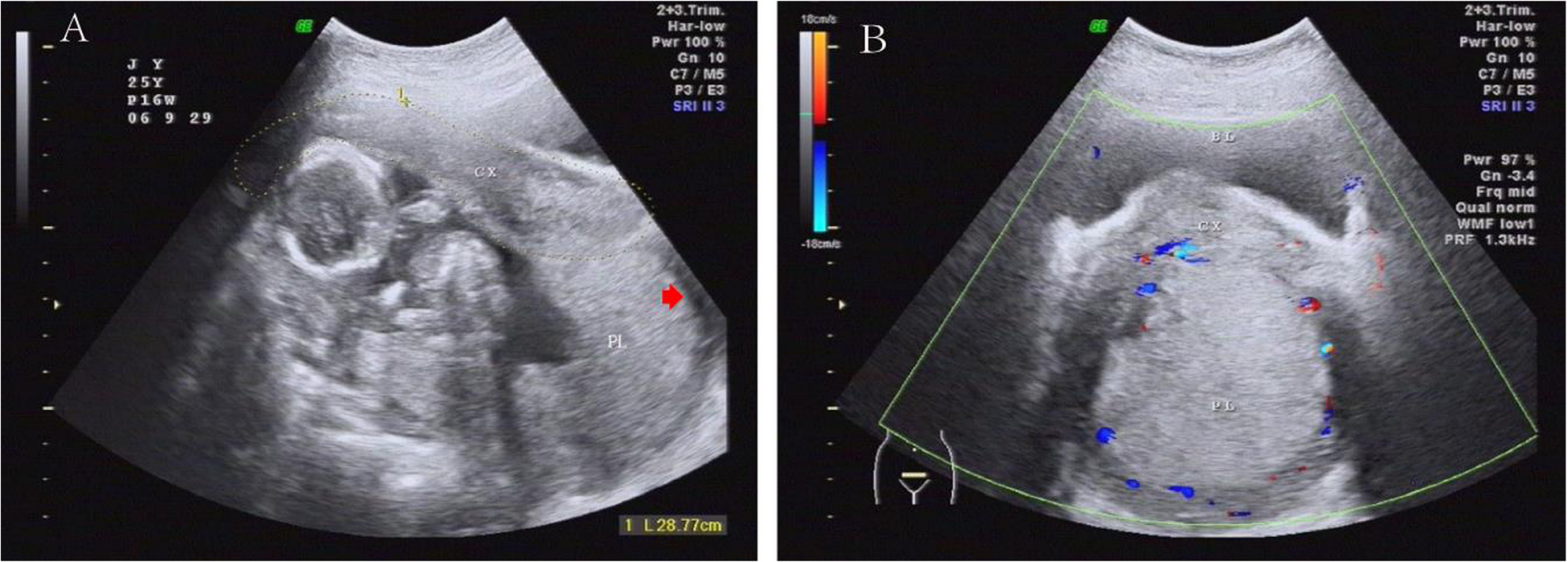
Ultrasonography of the retroverted gravid incarcerated uterus in the present case. (**a**) A longitudinal section shows that the uterus is fixed in retroversion with the cervix anteriorly transfixed behind the pubic symphysis above the uterine fundus (red arrow indicating the uterine fundus). Placenta located at fundus of uterus. (**b**) A transverse image of placenta that is located below the cervix. The two positional images better defines the position of uterine retroversion. BL: bladder; CX: cervix; PL: placenta

A repeated ultrasound scan in the tertiary hospital confirmed uterine retroversion with a fundus bending to the posterior fornix, which made the fundus into the lowest point of the uterus. The cervix was anteriorly transfixed behind the pubic symphysis and was barely above the fundus; fundal implantation of placenta was revealed (Fig. 1). A fetus with a heart rate of 156 bpm and adequate biometric measurements for the gestational age were observed. A Foley catheter was indwelt and 1075 mL of urine was emptied. Afterwards, the abdomen was soft to palpation. Pelvic examination revealed that the cervix could not be reached with fingers or exposed by vaginal speculum; the uterine fundus was palpated within the curvature of the sacrum, and there were occasional palpable uterine contractions without tenderness. Attempts to reduce incarceration by intravaginal pressure in the lithotomy position, in combination with the patient’s intermittent knee-chest position, were unsuccessful. However, incarceration was relieved by applying transvaginal fundal pressure in the second time. After the procedure, a 16-week-sized uterus was palpable, and the cervix was posterior in the vagina. Repeat ultrasound examinations confirmed that the uterus returned to the correct polarity. The patient could urinate without catheter. The pregnancy was subsequently uneventful, and a healthy female infant (3570 g, 50 cm, Apgar 9/10/10) was vaginally delivered at 38 weeks of gestation. (Case timeline see Additional file 1).

## Discussion and conclusions

Incarceration of the gravid uterus refers to the entrapment of the uterus in the pelvic cavity behind the sacral promontory. It has been estimated to affect 1 in 3000 pregnancies [2]. In addition to discuss our patient, we conducted a systematic search in the PubMed database, using the following search terms sequentially applied to all English reports published until 2016 (when the search was conducted): “(“retroverted uterus“ OR “retroverted gravid uterus“) AND (“incarceration” OR “incarcerated uterus” OR “incarcerated gravid uterus”) AND (“gestation” OR “gestational” OR “pregnant” OR “pregnancy” OR “gravid uterus”)”. The bibliographies of relevant articles were also searched by hand to identify additional eligible studies. (Additional file 2).

We identified 162 cases including our own for analysis (Additional file 3 and Additional file 4) [3–102]. The mean age of patients was 30.49 ± 5.66 years (16–42 years of age); the gestational age at the diagnosis of incarceration ranged from 5 weeks to 42 weeks; with 15.43% (25/162), 51.88% (83/162), and 28.13% (45/162) found in the first, second, and third trimesters of pregnancy, respectively (9 without specific information related to the time of disease onset). Thirteen cases were diagnosed at term pregnancy. Most women became pregnant through natural conception. Eight women became pregnant with ART, including 6 with IVF-ET and 2 with intracytoplasmic sperm injection (ICSI).

### Etiology and risk factors

The condition of a gravid uterine incarceration has no clearly identifiable causes, but is strongly correlated to malposition of the nonpregnant uterus, which is typically retroversion. In most cases, the gravid uterus transforms from a pelvic organ to an abdominal organ and the retroverted uterus corrects itself as the fundus rising out of the pelvis between 12 and 14 weeks of gestation and spontaneously falling forward to its normal anatomical position. On rare occasions, the uterus remains in a retroverted position and is trapped in the pelvic cavity. Multiple factors have been identified to prevent the uterus from entering the abdominal cavity, including tumor, uterine malformation, pelvic adhesions secondary to abdominal surgery, inflammation in the pelvis, and endometriosis. Among the 136 patients reviewed, 3 patients had uterine anomalies (didelphic uterus 2 [18, 69] and bicornuate uterus 1 [59]); 1 had abdominal surgery and presented with serious pelvic adhesion [52]; 1 had a deep sacral concavity [77]; and 1 had a history of cystitis [27]. Two patients reported no special history [62, 80] and the risk information was not available for 2 patients [33, 82]. Uterine prolapse, deep sacral concavity, and uterine fibroids are also identified as significant risk factors for a gravid uterus to develop incarceration [68, 77, 80]. It is noteworthy that there were 10 cases of recurrent incarceration [18, 27, 33, 52, 59, 62, 69, 77, 80, 82]. It appears that pregnant women who had experienced incarceration, especially those with known risk factors discussed above are likely to develop recurrent incarceration during the subsequent gestation.

Eight patients became pregnant through ART [63, 64, 66, 77, 82, 93] and carried significant risk factors for incarceration. There is no definitive report that associates incarceration in pregnant women with ART, but common risk factors associated with incarceration, such as endometriosis or pelvic inflammatory diseases, have also been identified for infertility. Gravid uterine incarceration should therefore be considered if a pregnant woman through ART develops abdominal pain and vaginal bleeding. Since woman with ART often receive more extensive monitoring during their pregnancies, gravid uterine incarceration may be diagnosed early and a timely manner, leading to fewer complications. In fact, 7 patients in sporadic case reports who successfully delivered infants because of prompt diagnosis through pelvic examination and abdominal imaging. Our patient had a history of lymphatic tuberculosis that could result in pelvic adhesion and bilateral tubal blockage, both of which could contribute to the development of uterine incarceration.

### Symptoms and diagnosis

Among the 162 reviewed cases, gravid uterine incarceration is mostly diagnosed in the second trimester. The symptoms of gravid uterine incarceration vary, but include urinary manifestations (53.70%, urinary retention, frequent urination, dysuria, urgency and paradoxical incontinence), abdominal pain (35.80%), constipation (6.79%), vaginal bleeding (6.17%), pelvic pain (6.79%), back pain (4.94%), tenesmus (1.85%), perineal pain (0.62%), and large painful mass prolapsed outside the anus (0.62%). Fourteen patients (8.64%) are asymptomatic, but also have delivered viable infants in the end, indicating that asymptomatic patients may better outcomes of pregnancy compared to those with severely symptomatic. Clinical complications usually occur after twelve weeks of gestation and are mostly related to the pressure from anatomical structures adjacent to the entrapped uterus, including lower abdominal and pelvic pain, dysuria, urinary frequency, urinary retention, overflow incontinence, rectal pressure, and worsening constipation [66, 92]. Among these, urinary retention is the most common symptom that occurs because of elongation of the urethra by displacement of the cervix, loss of the urethrovesical angle, and mechanical compression of the bladder neck. If an incarcerated uterus is not diagnosed and treated promptly, we speculate that bladder rupture, renal failure, spontaneous abortion, intra-uterine growth retardation, prematurity and premature rupture of the membranes, or even uterine sacculation or rupture may happen frequently.

Diagnosis of uterine incarceration remains difficult because its symptoms are often non-specific and absent in early pregnancy [82]. It is worth noting that in case reports, all diagnoses were made by clinical suspicion alone before 1969, but after 1974, especially after 2000, an increasing number of cases benefited from pelvic examination combined with imaging methods based on suspected symptoms. This could be explained by advances in imaging technology in recent years, which also indicated that ultrasound scanning or MRI facilitated early recognition and appropriate treatment of uterine incarceration.

In general, the features of pelvic examination can be described as follows. The cervix is anteriorly transfixed behind the pubic symphysis, making it difficult to expose. Additionally, sacculation of the posterior wall of the vagina and posterior fornix bulge may be observed, and the fundus is palpable within the curvature of the sacrum and could not be moved. Ultrasound examination could confirm incarceration. Abdominal sonography shows an advantage over transvaginal sonography in exhibiting the position of the cervix and its internal ostium and in determining the relationships between vagina, uterus and bladder, when the cervix is elongated and wedged behind the symphysis [80]. MRI is superior to ultrasound in the detailed scanning of gravid uterus incarceration [103]. It is suggested that every pregnant woman with an incarceration of the uterus should have MRI [104]. However, if the diagnosis is not suspected, the imaging findings can be misinterpreted as an intraperitoneal pregnancy, placenta previa or incorrect fetal presentation [88]. For our case, bloody vaginal discharge occurred at 15 weeks and 2 days of gestation, and urinary retention followed in the 16th week gestation. Even worse, this patient was misdiagnosed with a low position of the placenta based on an incorrect interpretation of the ultrasound scan in a local hospital.

### Treatment

No single treatment can be deemed more successful than the others for gravid uterus incarceration. Most obstetricians tend to replace the uterus in its natural position as soon as possible after diagnosis [80]. According to the gestational age, various management options may be considered. When incarceration of the retroverted gravid uterus is diagnosed in the late early trimester or early second trimester, obstetricians should fully evaluate the possibility of turning the uterus to a normal position. A passive reduction from a repeated knee-chest position after emptying the bladder can be recommended before 14 weeks of gestation. Between 14 and 20 weeks, the patient can also attempt a knee-chest position. If this method is unsuccessful, manual manipulation can be attempted. It is recommended to do this maneuver before 20 weeks of gestation, for more complications such as preterm labor may be caused by manual manipulation later than 20th week of gestation [105]. All maneuvers should only be performed after the bladder and bowel have been emptied, to reduce the risk of rupture of the bladder, bowel or uterus [48]. Additionally, pessary may be helpful after repositioning [1, 10, 19, 90]. Colonoscopic insufflation of the rectosigmoid at a gestational age of 13–15 weeks helped the reposition of the incarcerated uterus, which was reported by Seubert et al. [55]. It has been reported that the epidural anesthesia is an effective way to manage reduction of an incarcerated uterus [54]. In our literature review, six cases ended in a normal pregnancy after reposition under anesthesia [1, 27, 43, 83, 91, 92]. Anesthesia may increase the chance of a successful reduction because the uterus was easily released under anesthesia. If all interventions fail, laparoscopy or laparotomy is usually performed [66]. Operative procedures are not appropriate for patients with uterine incarceration, which is diagnosed before 20 weeks of gestation, because they can result in abortion or preterm delivery; furthermore, following such procedures, close follow-up is needed during the remainder of pregnancy. In the third trimester, uterine contractions usually fail to dilate the cervix because of incarceration. As a result, the risk of uterine rupture should be considered [85]. Caesarean section should be planned if reduction cannot be performed during the remainder of pregnancy [70, 78].

In 162 reviewed cases, cases of incarceration recognized in the first or second trimester of pregnancy account for 67.28% (109/162). Treatment of reposition was successfully attempted in 83 cases. After reposition, 68 patients successfully delivered infants [2, 15, 16, 19, 21, 23, 25, 27, 28, 36, 37, 41, 43, 44, 48, 50, 55, 62, 63, 66, 76, 77, 82, 83, 85, 88–92, 95, 98–101], including 36 term deliveries [16, 19, 21, 27, 28, 35, 37, 41, 43, 44, 48, 50, 62, 66, 76, 77, 87, 91, 98, 100, 101], and information for other cases was not available. Treatment methods vary in invasiveness, and because incarceration was quite rare, no study has yet been performed to determine the supremacy of any single treatment modality.

In the present case, the patient achieved correction from incarceration, which was presumed to be related to pelvic adhesions at 16 weeks, by manual manipulation, allowing for an attempt at repositioning. However, it could not be neglected that this patient had a past medical history that involved a high correlation with pelvic adhesions, which could prevent the gravid uterus from normal enlargement and ascent due to possible refractory incarceration. If this patient did not experience relief from incarceration via manual reposition, surgery with laparotomy or laparoscopy might be considered based on the patient’s strong desire to deliver a healthy child.

In conclusion, we report a case of gravid uterine incarceration with a history of lymphatic tuberculosis and IVF-ET. The particular risk factors, including a past history of infection and pregnancy by ART, made our case a relatively specific. As illustrated in the review of similar case reports, gravid uterine incarceration is a rare condition, but serious late gestational complications or poor obstetric outcomes may occur. Early diagnosis is the key to successful treatment. In view of the lack of specific signs or symptoms, additional physical and imaging examinations are critical to early diagnosis of this condition. Appropriate treatment measures that are tailored to the different gestation weeks may improve pregnancy outcomes.

## Supplementary information

**Additional file 1.** Case timeline. Describe the past medical history and interventions, summaries from initial and follow-up visits, diagnostic testing, interventions and relevant dates. (DOCX 17 kb)

**Additional file 2.** PRISMA flow diagram. Search terms sequentially applied to all English reports published until 2016 (when the search was conducted): “(“retroverted uterus“ OR “retroverted gravid uterus“) AND (“incarceration” OR “incarcerated uterus” OR “incarcerated gravid uterus”) AND (“gestation” OR “gestational” OR “pregnant” OR “pregnancy” OR “gravid uterus”)”. The bibliographies of relevant articles were also searched by hand to identify additional eligible studies. (DOC 33 kb)

**Additional file 3.** Case reports of incarceration of the retroverted uterus that have been reported in the literature (chronologically, up to 2016). There were 162 cases published, including the present case, based on all available articles which could be searched for in the PubMed database. Basic information of the patients and associated information from each case report worldwide were summarized for readers to obtain a general view on this condition, including possible risk factors, symptoms, diagnosis, treatment and outcomes. (DOCX 76 kb)

**Additional file 4.** Maternal characteristics with incarceration of the gravid uterus. Based on the 162 case reports, we conducted statistical analysis and summarized the maternal characteristics in the table. (DOCX 19 kb)

## Data Availability

All data generated or analysed during this study are included in this article.

## Abbreviations

ART: Assisted reproductive technology
BL: Bladder
CS: Caesarean section
CT: Computed tomography
CX: Cervix
G: Gravid
GA: Gestational age (weeks)
ICSI: Intracytoplasmic sperm injection
IVF-ET: In vitro fertilization and embryo transfer
LEEP: Loop electrosurgical excision procedure
MRI: Magnetic resonance imaging
NA: Not available
P: Partus
PL: Placenta
US: Ultrasound scan
