# Supplementary material for: Incarceration of the gravid uterus: a case report and literature review
Source: BMC Pregnancy Childbirth. 2019 Nov 8;19:408. doi: 10.1186/s12884-019-2549-3 (PMC6839127; doi:10.1186/s12884-019-2549-3)
Supplement: Supplementary file 1 — Additional file 1. Case timeline. Describe the past medical history and interventions, summaries from initial and follow-up visits, diagnostic testing, interventions and relevant dates. (DOCX 17 kb) [file 12884_2019_2549_MOESM1_ESM.docx]

| Dates | Relevant Past Medical History and Interventions | | |
| --- | --- | --- | --- |
| 2015.9.29 | Current Illness: A 25-year-old patient, gravida 1, para 0, first presented to the regional hospital at a gestation of 16 weeks and 1 day with a chief complaint of vaginal bloody discharge for 6 days, inability to urinate, and mild lower abdominal pain for half the day.  Past history: The patient experienced infertility for two years, which was confirmed by hystero-salpingo-graphy in the regional hospital, revealing bilateral obstruction of the fallopian tubes with uterine retroversion. As a result, she became pregnant by follicular aspiration and IVF-ET, and was treated since then with daily intramuscular progesterone. It should be noted that the patient was diagnosed with lymphatic tuberculosis at 17 years of age, but she had no history of sexually transmitted diseases, pelvic inflammatory diseases, endometriosis, uterine leiomyomas, deep sacral concavity, surgery, or congenital uterine malformations, such as uterus didelphys. | | |
| Dates | Summaries from Initial and Follow-up Visits | Diagnostic Testing  (including dates) | Interventions |
| 2015.9.23 | A small amount of bloody vaginal discharge persisted for one day. | an ultrasound report from the regional hospital revealed placenta almost covering the internal os of the cervix. | The patient was admitted to a gynecological ward with a diagnosis of a low position of the placenta and she continued Progesterone therapy. |
| 2015.9.29 | She experienced increasing lower abdominal pain and difficulty with urination | (2015.9.29)Reevaluation on ultrasound scan in our hospital confirmed uterine retroversion with a fundus bending to the posterior fornix. A fetus with a heart rate of 156 bpm and biometric measurements adequate for the gestational age were observed. | She was transferred to our tertiary hospital at gestational age 16 weeks and 1 day for further care.  A Foley catheter was indwelt and 1,075 mL of urine was emptied.  Pelvic examination.  Attempts to reduce incarceration by intravaginal pressure in the lithotomy position, in combination with the patient’s intermittent knee-chest position, were unsuccessful. Then, incarceration was relieved by applying transvaginal fundal pressure in the second time. |
| 2016.3.1 | A healthy female infant (3570g, 50 cm, Apgar 9/10/10) was vaginally delivered at 38 weeks of gestation. |  |  |
