# Supplementary material for: Incarceration of the gravid uterus: a case report and literature review
Source: BMC Pregnancy Childbirth. 2019 Nov 8;19:408. doi: 10.1186/s12884-019-2549-3 (PMC6839127; doi:10.1186/s12884-019-2549-3)
Supplement: Supplementary file 2 — Additional file 2. PRISMA flow diagram. Search terms sequentially applied to all English reports published until 2016 (when the search was conducted): “(“retroverted uterus“ OR “retroverted gravid uterus“) AND (“incarceration” OR “incarcerated uterus” OR “incarcerated gravid uterus”) AND (“gestation” OR “gestational” OR “pregnant” OR “pregnancy” OR “gravid uterus”)”. The bibliographies of relevant articles were also searched by hand to identify additional eligible studies. (DOC 33 kb) [file 12884_2019_2549_MOESM2_ESM.doc]

**Additional file 2 PRISMA Flow Diagram**

**Screening**

**Included**

**Eligibility**

**Identification**

Full-text articles assessed for eligibility
(n = 119 )

Full-text articles excluded, (n = 19 )

-Unmatched language(n=9)

-Articles without case reports (n=7)

- Incarceration of the non-gravid uterus (n=3)

Records identified through database searching
(n = 246 )

Additional records identified through other sources
(n = 12)

Records after duplicates removed
(n = 0 )

Records screened
(n = 258 )

Records excluded
(n = 139 )

Studies included in qualitative synthesis
(n = 100 )
