# Supplementary material for: Incarceration of the gravid uterus: a case report and literature review
Source: BMC Pregnancy Childbirth. 2019 Nov 8;19:408. doi: 10.1186/s12884-019-2549-3 (PMC6839127; doi:10.1186/s12884-019-2549-3)
Supplement: Supplementary file 3 — Additional file 3. Case reports of incarceration of the retroverted uterus that have been reported in the literature (chronologically, up to 2016). There were 162 cases published, including the present case, based on all available articles which could be searched for in the PubMed database. Basic information of the patients and associated information from each case report worldwide were summarized for readers to obtain a general view on this condition, including possible risk factors, symptoms, diagnosis, treatment and outcomes. (DOCX 76 kb) [file 12884_2019_2549_MOESM3_ESM.docx]

**Additional file 3 Case reports of incarceration of the retroverted uterus that have been reported in the literature (chronologically, up to 2016).**

| **Study** | **Age** | **Gravida/para** | **GA at diagnosis** | **The way of conception** | **Etiology, risk factors & possible related previous history** | **Symptoms** | **Diagnosis** | **Treatment** | **Outcome** |
| --- | --- | --- | --- | --- | --- | --- | --- | --- | --- |
| [3]Oldham (1859) | NA | NA | Term | Natural conception | None | NA | Pelvic examination | Term delivery | Infant nonviable |
| [4]Maiss (1899) | NA | NA | 3rd trimester | Natural conception | None | NA | Pelvic examination | Delivery at 3rd trimester with uterine rupture | Infant nonviable |
| [5]Stevens et al(1920) | 32y | NA | Very nearly a full-time pregnancy | NA | Bicornuate uterus; pelvic adhesions; a sacculation of the uterus itself formed by the greatly distended anterior wall | Laboring | Findings during CS | CS | Infant viable |
| [6]Dorman (1923) | NA | NA | Term | Natural conception | Bicornuate uterus | NA | Pelvic examination | Term delivery | Infant viable |
| [7]Oldfield (1931-1932) | NA | NA | Term | Natural conception | None | NA | Pelvic examination | Term delivery | Infant viable |
| [8]Murray et al (1934) | NA | NA | Term | Natural conception | None | NA | Pelvic examination | Term delivery | Infant viable |
| [9]Mendoza JT (1948) | NA | G1P0 | 24wk | NA | A flat pelvis with an overhanging sacral promotory | Labor; Urinary retention | Pelvic examination | CS | Infant nonviable |
| [10]Burdon S et al (1951) | 36y | P0 | 16wk | Natural conception | None | Urinary retention | Pelvic examination | Repositioning and pessary | No complications |
| [11]Seidner et al (1952) | 31y | P1 | 15wk | Natural conception | None | Urinary retention | Pelvic examination | Repositioning | No complications |
| [12]Spring et al (1952) | 29y | Multipara | 14wk | Natural conception | None | Urinary retention | Pelvic examination | Catheter×8d | No complications |
| [13]Freeth (1953) | 25y | G1P0 | 18wk | NA | An attack of cystitis two weeks before admission | Severe intermittent hypogastric pain with backache | Pelvic examination | Vaginal delivery, and the patient had aborted by rupturing the posterior wall of the cervical canal | Infant nonviable; a cervico-vaginal fistula formed and largely healed up later |
| [14] Ney et al (1954) | 30y | Multipara | 12wk | Natural conception | None | Urinary retention | Pelvic examination | Repositioning | No complications |
| [15]Devoe et al (1956) | 28y | P2 | 12wk | Natural conception | None | Urinary retention | Pelvic examination | Catheter×23d; spontaneous repositioning | Infant viable |
| [16]Terry (1959) | 30y | G3P2 | 10wk | Natural conception | NA | Perineal pressure during the 10th week, followed by constipation, frequent painful micturition and uterine bleeding | Pelvic examination | Repositioning in inverted decubitus position; term delivery | Infant viable |
| [17]Smith et al (1962) | 26y | G1P0 | 33wk | Natural conception | Bicornuate uterus; fibroid | Hydronephrosis | Pelvic examination | CS at 39wk; myomectomy the next year | Infant viable, 2700g |
| [18]Wood (1967) | NA | NA | 29wk | Natural conception | Didelphic uterus | Preeclampsia | Pelvic examination | Deliver at 29wk | Infant nonviable, 1163 g |
| [18]Wood (1967) | NA | NA | 30wk | Natural conception | Didelphic uterus | Recurrent incarceration | Pelvic examination | Deliver at 30wk | Infant nonviable, 1320 g |
| [19]Gibbons et al (1969) | 33y | G6P2 | 12wk | Natural conception | A history of appendectomy and right salpingectomy for tubal pregnancy | Urinary retention; paradoxical incontinence | Presenting with symptoms explainable by incarceration | Repositioning | Spontaneous abortion at 21wk |
| [19]Gibbons et al (1969) | 35y | G8P5 | 13wk | Natural conception | None | Urinary retention | Presenting with symptoms explainable by incarceration | Repositioning; pessary; term vaginal delivery | Infant viable |
| [19]Gibbons et al (1969) | 40y | G9P6 | 13wk | Natural conception | None | Urinary retention; abdominal pain | Presenting with symptoms explainable by incarceration; presenting with adherent retroversion | Repositioning; pessary; catheter; term vaginal delivery | Infant viable |
| [19]Gibbons et al (1969) | 25y | G2P1 | 13wk | Natural conception | None | Abdominal pain; urinary frequency; tenesmus | Presenting with symptoms explainable by incarceration | Repositioning; term vaginal delivery | Infant viable |
| [19]Gibbons et al (1969) | 36y | G4P3 | 14wk | Natural conception | None | Urinary retention | Presenting with symptoms explainable by incarceration | Repositioning; term vaginal delivery | Infant viable |
| [19]Gibbons et al (1969) | 27y | G1P0 | 16wk | Natural conception | None | Urinary retention; abdominal pain | Presenting with symptoms explainable by incarceration | Repositioning (failed); laparotomy & suspension; term vaginal delivery | Infant viable |
| [19]Gibbons et al (1969) | 26y | G3P1 | 16wk | Natural conception | A history of appendectomy | Abdominal pain; staining | Presenting with symptoms explainable by incarceration | Repositioning (anesthesia) | Spontaneous abortion 4hr. postopartum |
| [19]Gibbons et al (1969) | 21y | G1P0 | 19wk | Natural conception | A simple ovarian cyst | Abdominal pain; dysuria; constipation; staining | Presenting with symptoms explainable by incarceration | Abdominal hysterotomy | Abortion |
| [19]Gibbons et al (1969) | 24y | G3P2 | 20wk | Natural conception | None | Abdominal pain; staining | Presenting with symptoms explainable by incarceration | Repositioning | Spontaneous abortion in 12hr. |
| [20]Fadel et al (1974) | 19y | G4P1 | 25wk | Natural conception | A history of right salpingo-oophorectomy for tubal pregnancy and a spontaneous abortion at 14 wk | Lower abdominal pain, urinary frequency and dysuria from 14th week | Amniogram; findings during CS | CS | Infant nonviable, 725g |
| [21]Swartz et al (1977) | 30y | G1P0 | 17wk | Natural conception | Bladder atony; postobstructive diuresis | Urinary retention | Pelvic examination | Repositioning; term delivery | Infant viable |
| [22]Spearing (1978) | NA | G2P0 | 39wk | Natural conception | Unicornuate uterus | Spontaneous rupture of membranes and infrequent contractions | Pelvic examination | CS at 39wk | Infant viable, 3860g |
| [23]Laing(1981) | 22y | G3P2 | 17wk | NA | Recurrent urinary tract infecions | None | US | Repositioning | Infant viable |
| [23]Laing(1981) | 27y | G1P0 | 12wk | NA | None | None | US | Repositioning | Infant viable |
| [24]Kondo et al (1982) | 27y | G1P0 | 19wk | NA | A history of 6 operations for removal of polypoid tumor in the small intestine because of Peutz-Jeghers syndrome | Intermittent urinary retention and difficulty in urination for 2wks | Pelvic examination and cystoscopy | Term delivery at 37wk without self-catheterization | Infant viable |
| [25]Chatterjee (1984) | NA | NA | 42wk | Natural conception | None | NA | Pelvic examination | Induction of labor | Intrauterine fetal death at 42wk |
| [25]Chatterjee (1984) | NA | NA | Term | Natural conception | None | Partial uterine rupture | Pelvic examination | CS and subtotal hysterectomy | Infant viable |
| [26]Nelson (1986) | 30y | G4P2 | 14wk | Natural conception | NA | Lower abdominal pain; urinary frequency and urgency; nocturia; constipation; urinary retention | Pelvic examination | Take ampicillin; repositioning | An uneventful pregnancy |
| [27]Silva et al (1986) | 27y | G2P1 | 12wk | Natural conception | A history of similar symptoms at 14wk of last pregnancy but spontaneously resolved days later; two episodes of uncomplicated cystitis during childhood | Marked suprapubic pain; urgency; urinary retention | Pelvic examination | Repositioning; term delivery | Infant viable |
| [27]Silva et al (1986) | 34y | G2P1 | 12wk | Natural conception | None | Agonizing suprapubic pain; abdominal distension; urinary retention | Pelvic examination & US | Repositioning (anesthesia); term delivery | An episode of uncomplicated cystitis; infant viable |
| [28]Evans AJ et al (1986) | 20y | G3P1 | 37wk | Natural conception | A spontaneous abortion ar 24wk during the first pregnancy; CS complicated by a major placenta preaevia with a 2540g infant; bicornuate uterus | Asymptomatic | Pelvic examination | Repositioning & CS | Infant viable, 2860g |
| [29]Schwartz et al(1986) | 39y | G3P2 | 7wk | NA | A large leiomyoma | Acute urinary retention | US | Cystoscopy; Laparotomy; Hysterectomy | Recovery |
| [30]Edminster et al (1987) | 28y | G7P3 | 12wk | Natural conception | None | Urinary frequency; tenesmus; abdominal pain; vaginal spotting | Pelvic examination | Repositioning & curettage | Follow up with her hometown physician |
| [31]Meislin (1987) | 26y | G2P1 | 14wk | Natural conception | None | Lower abdominal pain; vaginal discharge | Pelvic examination & US | Repositioning | Total relief and nonrecurrence of symptoms; routine follow-up by her obstetrician. |
| [32]Vleugels et al(1987) | 24y | G1P0 | 37wk | NA | A uterus subseptus. | Abdominal pains at 28wk which disappeared spontaneously; laboring | Reveal uterine retroversion during examination of the abdominal organs in surgery | CS | Infant viable, 2070g |
| [33]McGann et al (1988) | NA | G1 | 31wk | Natural conception | NA | Preeclampsia | Pelvic examination | Deliver at 32wk | Infant viable |
| [33]McGann et al (1988) | NA | G2 | 32wk | Natural conception | NA | Recurrent incarceration | Pelvic examination | Deliver at 33wk | Infant viable |
| [34]Jackson et al (1988) | 24y | G2P1 | 36wk | Natural conception | None | Asymptomatic | Reveal uterine retroversion during examination of the abdominal organs in surgery | CS at 36wk | Infant viable |
| [35]Hess et al (1989) | 36y | P2 | 14wk | Natural conception | None | Urinary retention | Pelvic examination | Repositioning; term delivery | Infant viable |
| [35]Hess et al (1989) | 32y | P1 | 17wk | Natural conception | None | Urinary retention | Pelvic examination | Repositioning; term delivery | Infant viable |
| [35]Hess et al (1989) | 39y | P1 | 12wk | Natural conception | None | Urinary retention | Pelvic examination | Repositioning; term delivery | Infant viable |
| [35]Hess et al (1989) | 25y | P1 | 15wk | Natural conception | None | Urinary retention | Pelvic examination | Repositioning; term delivery | Infant viable |
| [36]Hankins et al(1989) | 39y | multigravida | 13wk | Natural conception | With a large posterior leiomyoma in uterus | Suprapubic pain, urinary hesitancy, frequency, and small urinary voiding volumes | Sonographic evaluation | A Foley catheter was placed; Repositioning under the help of knee-chest position and drainage by Foley catheter; term delivery | Infant viable |
| [37]Smalbraak et al (1991) | 31y | G1 | 15wk | Natural conception | NA | Lower abdominal pain; increasing urinary frequency, hesitance and a sense of residual urine, especially at night from 14th week | Pelvic examination & US | Repositioning; a micturition regimen (i.e., micturition at least every 4h) prescribed for 2 wk; term delivery | Infant viable |
| [37]Smalbraak et al (1991) | 28y | G1 | 35wk | Natural conception | NA | Lower abdominal pain and urinary retention between 12th and 16th week; a serious rise in blood pressure at 35 wk; urinary retention at 36 wk | Pelvic examination & US | Spontaneous repositioning at 36wk; CS | Infant viable, 3035g |
| [37]Smalbraak et al (1991) | 26y | G2P0 | 16wk | Natural conception | NA | Asymptomatic | Pelvic examination & US | Repositioning; CS at 34wk | Infant viable, 1220 g |
| [37]Smalbraak et al (1991) | 34y | G1 | 36wk | Natural conception | NA | Severe low abdominal pain, urinary retention and diarrhea between 14th and 18th week, disappearing spontaneously after 18 wk | Pelvic examination & US | CS | Infant viable, 2830 g |
| [38]Van Winter et al (1991) | 34y | G4P0 | 35wk | Natural conception | Bicornuate uterus | Fundal height failed to increase at 35wk; regular contractions accompanied by rectal pain at 39wk | Pelvic examination | CS | Infant viable |
| [38]Van Winter et al (1991) | 32y | G2P1 | 26wk | Natural conception | A vaginal delivery at 36wk with an adherent placenta that necessitated removal with general anesthesia; a bicornuate uterus | Severe low back pain; hip pain; a rectovaginal mass | Pelvic examination & US & MRI | CS | Infant nonviable, 860g |
| [38]Van Winter et al (1991) | 33y | G2P1 | 15wk | Natural conception | An ovarian cystectomy and appendectomy 11 years ago; abdominoplasty due to dense pelvic adhesions 5 years ago | Asymptomatic; premature rupture of membranes | US | CS | Infant viable, 1935g |
| [39]Keating et al (1992) | 19y | G1P0 | 34wk | Natural conception | Bicornuate uterus | Hypertension and edema from 30th week; hydronephrosis with dilatation of the upper right ureter from US of kidneys | Reveal incarceration during examination of the abdominal organs in surgery | CS; hysterectomy; bladder repaired; catheter left in situ for 10d | Infant viable |
| [40]Gunn (1993) | 28y | G3P0 | 28wk | Natural conception | None | Asymptomatic; premature rupture of membranes at 28wk | Reveal incarceration during examination of the abdominal organs in surgery | CS | Infant viable |
| [41]Hill et al(1993) | 30y | G2P1 | 14.6wk | Natural conception | A history of CS; a heartshaped uterus with a known transverse septum | Asymptomatic | US | Spontaneous repositioning at 26wk; spontaneous vaginal delivery at 37.7wk | Infant viable, 2880g |
| [42]Nwosu et al(1993) | 20y | G1P0 | 23wk | NA | Bicornuate uterus (pregnancy in an incarcerated sacculated non-communicating rudimentary horn) | Low abdominal pain and recurrent urinary retention | Laparotomy | CS | Infant viable, 444g |
| [43]Lettieri et al (1994) | 36y | G1P0 | 31wk | Natural conception | None | Preterm Labor; placenta previa | Pelvic examination | CS at 31wk | Preterm delivery; infant viable |
| [43]Lettieri et al (1994) | 23y | G2P0 | 14wk | Natural conception | None | Urinary retention; pelvic pressure | Pelvic examination | Repositioning via laparoscopy at 15wk; term vaginal delivery | Infant viable |
| [43]Lettieri et al (1994) | 34y | G8P1 | 14wk | Natural conception | None | Pelvic pain; constipation | Pelvic examination | Repositioning (manually on 2nd attempt); CS at term | Infant viable |
| [43]Lettieri et al (1994) | 35y | G3P1 | 13wk | Natural conception | None | Urinary retention | Pelvic examination | Repositioning (anesthesia) at 14wk; CS at term | Infant viable |
| [43]Lettieri et al (1994) | 40y | G1P0 | 14wk | Natural conception | None | Pelvic pressure | Pelvic examination | Repositioning; term vaginal delivery | Infant viable |
| [43]Lettieri et al (1994) | 42y | G5P3 | 14wk | Natural conception | None | Complete urinary retention; pelvic pain | Pelvic examination | Bladder drained with Foley catheter; repositioning; term vaginal delivery | Infant viable |
| [43]Lettieri et al (1994) | 31y | G7P1 | 13wk | Natural conception | None | Pelvic pain; urinary symptoms; constipation | Pelvic examination | Repositioning via laparoscopy at 14wk; term vaginal delivery | Infant viable |
| [44]Wittich et al (1994) | 30y | G2P0 | 11wk | Natural conception | A 9×9cm right corneal myoma | Increasing pelvic and low back pain, urinary frequency and urgency, and tenesmus | US | Repositioning through laparotomy under general endotracheal anesthesia without myomectomy; low forceps delivery at 41wk | Infant viable, 3290g |
| [45]Emery et al(1994) | 27y | G6P5 | 12wk | Natural conception | None | Suprapubic pain and difficuity voiding. | Pelvic examination & US | Bladder drained with urinary catheter; repositioning by manipulation | The patient had no difficulty  voiding and was discharged  home in good condition. |
| [46]Myers et al(1995) | 30y | G3P1 | 13.5wk | NA | NA | Urinary retention and dysuria | Pelvic examination | Manual repositioning failed; teach the patient the knee-chest position, and spontaneous repositioning by 18wk | NA |
| [47]Renaud et al (1996) | 30y | G2P0 | 36wk | Natural conception | A history of ectopic pregnancy with methotrexate treatment | Preeclampsia | Reveal incarceration during examination of the abdominal organs in surgery | CS | Infant viable |
| [48]Patterson et al (1997) | 22y | G1P0 | 12wk | Natural conception | A history of an uncomplicated appendectomy at age 11 | severe perineal pain and an urge to void | Pelvic examination & US | Catheter; spontaneous repositioning the next day; term vaginal delivery | Infant viable |
| [49]Feusner et al (1997) | 32y | G2P0 | 14wk | Natural conception | Fibroid uterus and an elective first-trimester abortion 6 years ago complicated by infection | intermittent, crampy, lower abdominal pain | Pelvic examination | Repositioning | Follow-up |
| [50]Hirsch(1997) | 21y | G3P0 | 17wk | NA | One hospitalization for pelvic inflammatory disease; Chlamydia trachomatis infection | Lower abdominal pain | Pelvic examination | Repositioning for anterior uterine incarceration; treatment for Chlamydia; term delivery | Infant viable |
| [51]Dietz et al (1998) | 33y | G3P0 | 32^+^wk | Natural conception | A history of a left salpingectomy for an ectopic pregnancy and a right ovarian cystectomy | 1 episode of spotting at 16wk; severe lower abdominal pain at 32^+5^wk | MRI | CS at 32^+6^wk | Infant viable; uterine involution being found on day 4 by US |
| [52]Jacobsson et al (1999) | 32y | G3P1 | 29wk | Natural conception | At 25y, a healthy boy through CS of the first pregnancy with 33wk; at 27y, an extrauterine pregnancy in the left tube and a tubotomy, with adhesions from the uterine fundus to the abdominal wall; a history of two years of secondary infertility by a diagnostic laparoscopy with new adhesions from the abdominal wall to the left tube and the sigmoid colon; no passage through the left tube by methylene blue exam | Abdominal pain during the second trimester | Pelvic examination & history | CS at 34^+6^wk | Infant viable |
| [53]O'Connell et al (1999) | 27y | G1P0 | 25wk | Natural conception | None | Severe abdominal pain and both kidneys remained hydronephrotic despite the insertion of bilateral ureteric stents | Pelvic examination & MRI | A left nephrostomy; CS due to pyrexia at 28wk | Infant viable; second pregnancy 18 months later and premature membranes ruptured at 33wk, delivery with a male infant by CS |
| [54]Algra et al (1999) | 30y | G1P0 | 12^+3^wk | Natural conception | A uterine fibroma | Pelvic pain | Pelvic examination | Repositioning (anesthesia) | Discharged home |
| [54]Algra et al (1999) | 33y | G5P3 | 10^+4^wk | Natural conception | None | Lower quadrant pain; urinary retention | Pelvic examination | Repositioning (anesthesia) | Discharged home |
| [55]Seubert et al(1999) | NA | NA | 5 cases with 13-15wk | NA | NA | NA | NA | Repositioning by a colonoscopy-assisted manipulation in all 5 cases | No pregnancy losses occurred after the procedure in all 5 cases |
| [56]Love et al (2000) | 33y | G4P1 | 14wk | Natural conception | None | Severe abdominal pain & urinary retention | US | Catheter & Repositioning | Normal |
| [56]Love et al (2000) | 27y | G5P4 | Uncertain but early  in the third month | Natural conception | None | Frequency and burning with urination; severe distress from lower abdominal and back pain | Pelvic examination | Catheter | Normal |
| [57]DeFriend et al (2000) | 33y | G3P2 | 32wk | Natural conception | A history of CS in the first pregnancy; a ventouse-assisted delivery in the second | Abdominal pain and difficulty in passing urine | MRI | CS; total abdominal hysterectomy due to the placenta percreta | Infant viable |
| [58]Li et al (2000) | 37y | G1P0 | Term | Natural conception | A history of anterior intramural myomectomy 2 years ago; significant adhesions between the anterior fundal wall with the omentum and sigmoid colon | Asymptomatic | Reveal incarceration during examination of the abdominal organs in surgery | An elective CS; the uterus was returned to a normal position by manual extraction of the retroverted fundus; lysis of the adhesion | Infant viable, 3600g |
| [59]Minassian et al (2000) | 24y | G3P0 | ＜12wk | Natural conception | Bicornuate uterus | Urinary retention | Pelvic examination | US guided curettage | None |
| [59]Minassian et al (2000) | 24y | G4P0 | 33wk | Natural conception | Bicornuate uterus | Urinary retention | Pelvic examination | Vaginal delivery | Infant viable, 1715 g |
| [2]Hamod et al (2002) | 24y | G1P0 | 18wk | Natural conception | Fibroid on the anterior wall | Urinary retention | US & MRI | Catheter & intermittent self-catheterisation after discharge; conservative treatment unitl 36wk, when performing CS and removing the fibroid; spontaneous repositioning just before the CS | Correction of uterine position and infant viable |
| [60]Yohannes et al (2002) | NA | G2P1 | 15wk | Natural conception | None | Urinary retention | Pelvic examination & history | Catheter & Repositioning | No urologic complications for the remainder of the pregnancy. |
| [61]Uma et al (2002) | 35y | G1P0 | 32wk | Natural conception | Multiple fibroids | Severe abdominal pain and pain in her left calf at 26wk; left leg swollen at 30wk | Reveal incarceration during examination of the abdominal organs in surgery | Corticosteroids; CS at 32wk; catheter; hysterectomy | Infant viable |
| [62]van Beekhuizen et al (2003) | 20y | G1P0 | 18wk | Natural conception | None | A small amount of vaginal bleeding and abdominal pain | MRI | Repositioning; delivery after membranes ruptured | Infant viable, 180g |
| [62]van Beekhuizen et al (2003) | 18y | G5P1 | 36wk | Natural conception | Didelphic uterus and vaginal septum; two spontaneous abortions | Incomplete voiding at 21wk and resolving without therapy; abdominal discomfort at 33wk; increasing abdominal pain and voiding and defecation problems at 36wk | MRI | Repositioning & CS at 37wk | Infant viable, 2800g |
| [62]van Beekhuizen et al (2003) | 40y | G1P0 | 23wk | Natural conception | Bicornuate uterus; a history of endoscopic removal of submucous leiomyomas | NA | Intrauterine death at 23wk; MRI confirmed incarceration | Repositioning (failed) | Expulsion of a normal fetus in breech position; operative removal of four leiomyomas 3 months later; uneventful second pregnancy by CS |
| [62]van Beekhuizen et al (2003) | 28y | G1P0 | 22wk | Natural conception | None | Abdominal pain and discomfort while urinating | MRI | Repositioning at 22wk; a recurrence of incarceration one week later; spontaneous repositioning 3 days later; CS at 32wk | Infant viable, 1474g. |
| [62]van Beekhuizen et al (2003) | 33y | G1P0 | 28wk | Natural conception | Two leiomyomas | Abdominal tenderness at 16wk and relief with epidural analgesia; premature contractions at 25wk and relief with tocolysis; intrauterine death occurred at 28wk | MRI | Repositioning (failed) & amniocentesis & CS | Infant nonviable with multiple congenital anomalies; the initially incarcerated leiomyoma removed; uneventful second and third pregnancy by CS |
| [63]Childs et al (2003) | 35y | G1P0 | 9wk | IVF-ET | A history of endometriosis | inability to urinate and mild abdominal pain at 9wk; acute urinary retention | US | Repositioning; CS at 34wk | Infants viable (triplets) |
| [64]Matsushita et al (2004) | 33y | G2P0 | 40wk | IVF-ET | A history of the neonatal repair of an omphalocele; left tubectomy 2 years previously due to left tubal pregnancy following IVF-ET; being treated with danazol for endometriosis at 21y; an adhesion between the posterior wall of the uterus and the pouch of Douglas being found during this CS | Asymptomatic | Pelvic examination & MRI | CS | Infant viable, 3552g |
| [65]Yang et al(2004) | 35y | G1P0 | 12wk | NA | NA | Acute urinary retention | Pelvic examination & US | Bladder drained with urinary catheter; repositioning by manipulation | No further voiding problems |
| [65]Yang et al(2004) | 28y | G2P1 | 12wk | NA | NA | Acute urinary retention | Pelvic examination & US | Bladder drained with urinary catheter; repositioning by manipulation | No further voiding problems |
| [65]Yang et al(2004) | 25y | G1P0 | 14wk | NA | NA | Acute urinary retention | Pelvic examination & US | Bladder drained with urinary catheter; repositioning by manipulation | No further voiding problems |
| [65]Yang et al(2004) | 30y | G2P1 | 11wk | NA | NA | Acute urinary retention | Pelvic examination & US | Bladder drained with urinary catheter; repositioning by manipulation | A repeat episode of urinary retention and being resolved by 16th week |
| [65]Yang et al(2004) | 37y | G2P1 | 12wk | NA | NA | Acute urinary retention | Pelvic examination & US | Bladder drained with urinary catheter; repositioning by manipulation | No further voiding problems |
| [66]Inaba et al (2005) | 27y | G1P0 | 15wk | IVF-ET | Closed uterine tube revealed by hysterosalpingography | Urinary retention from 14wk | Pelvic examination & US | Repositioning; CS at 38wk | Infant viable |
| [66]Inaba et al (2005) | 32y | G3P0 | 14wk | IVF-ET | Endometriosis (class IV of Beecham classification) since 25y, with closed tubes; left salpingectomy due to ectopic pregnancy at first; being pregnant after the fourth IVF-ET; adhesions between the pouch of Douglas and the uterine fundus during this CS | Urinary retention from 14wk | Pelvic examination & MRI | Urethral catheterization; Repositioning (failed); CS at 34wk | Infant viable, 2094g |
| [67]Frei et al (2005) | 34y | G2P1 | 29wk | Natural conception | A known history of retroverted uterus | Recurrent pain and constipation | US & MRI | Bilateral nephrostomas being placed; CS at 34^+1^wk; the nephrostomas being removed | Infant viable, 1835g |
| [68]Ozel(2005) | 40y | G4P2 | 19wk | NA | 2 CS; 2 subsequent spontaneous abortions; uterine prolapse | Inability to void; progressive constipation | Pelvic examination & US | A Foley catheter; 2 manual reductions unsuccessful; successful manual repositioning under epidural anesthesia | NA |
| [69]Sutter et al (2006) | 34y | G1 | 29wk | Natural conception | Uterus didelphys, hypoplastic right uterus | Abdominal tenderness | MRI | Vaginal delivery | Infant viable, 1715 g |
| [69]Sutter et al (2006) | 34y | G2P1 | 32wk | Natural conception | Uterus didelphys, hypoplastic right uterus | Abdominal tenderness | MRI | CS | Infant viable |
| [70]Singh et al (2007) | 23y | G1P0 | 33wk | Natural conception | None | Generalized abdominal pain; a heavy sensation in the vagina, frequency of micturition and nocturia at 27wk; severe lower back and abdominal pain at 33wk | Pelvic examination & MRI | CS | Infant viable; 2140g; pulmonary embolism in thirty-six hours after delivery, with therapy of heparin |
| [71] Barton-Smith et al(2007) | NA | NA | 38wk | NA | A reduced anteroposterior diameter, a reduced sacral curve in the upper pelvis, and tapering of the lower body of S1 | NA | Reveal incarceration during examination of the abdominal organs in surgery | CS | Infant viable, 1900 g |
| [72]Chauleur et al (2008) | 33y | G2P0 | 5wk | Natural conception | A history of a posterior myometrial fibroid 3 years ago | Acute urine retention at 5wk | Pelvic examination | Bladder catheterization; repositioning (failed); a laparotomic myomectomy; CS at 37 wk | Infant viable, 2470g |
| [72]Chauleur et al (2008) | 41y | G1P0 | 6wk | Natural conception | A posterior intramural fibroid | Urinary retention | Pelvic examination | Bladder catheterization; repositioning (failed); myomectomy | Abortion 1wk later; a second pregnancy 6 months later, infant viable 2820 g by CS at 37wk |
| [1]Sweigart et al (2008) | 29y | G6P1 | 12wk | Natural conception | Endometriosis with four laparoscopic procedures; an adenexal cyst; four previous miscarriages; a prior CS, and Perry-Romberg syndrome (localized scleroderma of childhood). | Fever, sacral pain and urgency | Pelvic examination | Repositioning (anesthesia); pessary | The pregnancy progressed |
| [73]Charova et al (2008) | 33y | G1P0 | 31wk | Natural conception | None | Abdominal discomfort from 19wk; lower abdominal pain, frequency of micturition between 29 and 30 wk; at 31 wk, with intermittent period-type lower abdominal pain, backache and confirmed rupture of membrane | Reveal incarceration during examination of the abdominal organs in surgery | CS | Infant viable |
| [74]Gottschalk et al (2008) | 16y | G1P0 | 24wk | Natural conception | A history of retroverted uterus | Intermittent severe bilateral flank pain at 24 wk | Pelvic examination & MRI | CS at 35^+2^wk | Infant viable |
| [75] Lee et al(2008) | 28y | G1P0 | 17wk | NA | A large uterine myoma | Vaginal leakage and urinary retention; rupture of membranes | Pelvic examination & US & MRI | A Foley catheter was inserted; termination of pregnancy by CS; after CS, receive four cycles of GnRHa and myomectomy 6 months later | Infant nonviable,160 g |
| [76]Rose et al(2008) | 30y | G1P0 | 15wk | NA | 3 uterine myomas | Urinary retention | US | A Foley catheter; spontaneous reduction at 20wk following 3 times unsuccessful manual reductions; CS at 40wk | Infant viable |
| [77]Hooker et al (2009) | 35y | G1P1 | 29wk | ICSI | A long period of infertility; being exposed to diethylstilbestrol; deep sacral concavity with prominent promontorium | Being colicky pain at the left side because of hydronephrosis | Pelvic examination & US | A nephrostomy catheter was placed percutaneously at 30wk; CS at 38wk | Infant viable; being pregnant again spontaneously 2 years later, with a recurrent incarcerated retroverted gravid uterus at 14wk, and repositioning through a laparotomy at 16wk, with an infant viable by CS at 38wk |
| [78]van der Tuuk et al (2009) | 35y | G1P0 | 37^+6^wk | Natural conception | None | Spontaneous rupture of membranes and loss of amniotic fluid | Reveal incarceration during examination of the abdominal organs in surgery | CS | Infant viable; a second pregnancy 1 year later, with a uterus in retroversion at 18 weeks asymptotic; delivery with a live infant at 37^+^5wk |
| [79]Gerscovich et al(2009) | NA | NA | 12wk | NA | NA | Vaginal spotting | US | NA | NA |
| [80]Dierickx et al (2010) | 34y | G1P0 | 32wk | Natural conception | None | Pulmonary edema and renal  failure at 32wk | Reveal incarceration during examination of the abdominal organs in surgery | CS | Infants nonviable (twins); a second pregnancy 2 years later, with incarceration at 18wk confirmed by MRI; infant viable at 37wk by CS |
| [81]Al Wadi et al (2011) | 30y | G1P0 | 18wk | Natural conception | Uterus didelphys | Asymptomatic | US & MRI | CS | Infant viable |
| [82]Dierickx et al (2011) | 39y | G1P0 | 22wk | ICSI | Endometriosis | Urine urgency | MRI | Colonoscopy-assisted repositioning; vaginal delivery | Infant viable |
| [82]Dierickx et al (2011) | 29y | G1P0 | 22wk | Natural conception | Deep sacral concavity with prominent promontorium | Dysuria and incomplete bladder emptying | MRI | Colonoscopy-assisted repositioning failed; repositioning by laparotomy; CS | Infant viable |
| [82]Dierickx et al (2011) | 28y | G3P2 | 15wk | Natural conception | None | Urinary retention | Pelvic examination & history | Colonoscopy-assisted repositioning; vaginal delivery | Infant viable |
| [82]Dierickx et al (2011) | 36y | G2P1 | 18wk | Natural conception | Recurrent incarceration | Urinary retention | MRI | Colonoscopy-assisted repositioning; CS | Infant viable, 1820g |
| [83]Grossenburg et al (2011) | 33y | G3P1 | 21wk | Natural conception | Hypothyroidism and ulcerative colitis; colectomy with diverting ileostomy and creation of a pouch for an ileal-anal pull-through followed by subsequent takedown of the ileostomy | Urinary retention | Pelvic examination & MRI | Repositioning(US guidance & anesthesia);vaginal delivery | Infant viable, 2300g |
| [84] Al Wadi et al (2011) | 30y | G1P0 | 22wk | Natural conception | Uterus didelphys | Asymptomatic | US & MRI | CS at 38wk | Infant viable |
| [85]Wang et al (2012) | 25y | G2P0 | 18wk | Natural conception | Adhesions between the fundus of uterus and the pouch of Douglas were found in the process of surgery | Asymptomatic | Pelvic examination & US | Repositioning; termination of pregnancy was performed by hysterotomy delivery | Successful |
| [86]Hachisuga et al (2012) | 38y | G1P0 | 29^+4^wk | Natural conception | 2 uterine leiomyomas | Spontaneous rupture of membranes | Pelvic examination & US & MRI | CS | Infant viable, 1240g |
| [87]Shima et al(2012) | 37y | G1P0 | 24wk | NA | A huge myoma was next to the internal os. | Intermittent uterine contractions | MRI | Uterine contractions were controlled by administration of ritodrine hydrochloride at 29wk; elective cesarean section was performed under general anesthesia at 37wk | Infant viable |
| [88]Fernandes et al(2012) | NA | NA | NA | NA | 1 of 8 cases with uterine didelphys and asymptomatic incarceration in a previous pregnancy; 2 of 8 cases with large posterior/fundal fibroids | 3 of 8 cases with abdominal or pelvic pain alone; 4 of 8 cases with urinary retention | US & MRI | 4 of 8 cases with surgical reduction; 1 with spontaneous reduction | Infant viable in 7 of 8 cases; infant nonviable in 1 of 8 cases |
| [89]Katopodis et al(2013) | NA | Nulliparous | 13wk | NA | Leiomyoma | None | US & MRI | Conservative management after unsuccessful manual reduction; spontaneous reduction following degeneration of the fibroid at 25wk | Infant viable, 4163g |
| [90]Dierickx et al (2014) | 35y | G1P0 | 17wk | Natural conception | None | Urinary retention and abdominal pain | Pelvic examination & US & MRI | Bladder catheterization; repositioning by a colonoscopy-assisted manipulation; a pessary; vaginal delivery at 39wk | Infant viable, 2935g |
| [91]Policiano et al (2014) | 34y | G1P0 | 21wk | Natural conception | A history of two hepatic transplantations | Abdominal pain, constipation and dysuria; asymptomatic at 30wk | Pelvic examination & US | Spontaneous repositioning; term vaginal delivery | Infant viable, 2825g |
| [91]Policiano et al (2014) | 31y | G1P0 | 15wk | Natural conception | None | Increasing abdominal pain and increased urinary frequency | Pelvic examination | Repositioning (anesthesia) at 18wk; CS at 37wk | Infant viable, 2265g |
| [92]Newell et al (2014) | 41y | G1P0 | 18wk | Natural conception | None | Urinary retention and constipation at 17wk; worsening abdominal pain and bloating at 20wk; bilateral hydronephrosis | US | Rectal insufflation and manual disimpaction under general anesthesia at 22 6/7 wks | The amniotic membranes spontaneously ruptured and intrauterine death 12 hours after the uterus was reduced, delivery |
| [92]Newell et al (2014) | 33y | G3P2 | 15wk | Natural conception | A history of vaginal delivery at term followed by an emergency caesarean delivery at 32 weeks of gestation for a placental abruption | Urinary retention; severe abdominal pain | US | Catheter; Repositioning (anesthesia); CS | Infant viable, |
| [93]Matsushita et al(2014) | 36y | G1P0 | 16^+4^wk | IVF-ET | None | Urinary retention | MRI | Catheter; failed repositioning; intrauterine fetal death at 21wk and induced labor after successfully reposition | Infant nonviable, 155 g |
| [94]Slama et al(2015) | 37y | G6P2 | 13wk | NA | None | Urinary retention, bilateral costo-vertebral tenderness and thick white discharge on  speculum exam | US | Self-catheterization,  Pessary | NA |
| [94]Slama et al(2015) | 42y | G5P3 | 13wk | NA | Infertility, LEEP, salpingectomy | Urinary retention | US | Self-catheterization,  pessary placement | NA |
| [94]Slama et al(2015) | 22y | G15P5 | 21wk | NA | Multiple spontaneous abortions, clotting disorder | Urinary retention, left upper quadrant pain, nausea | US | Pessary placement | NA |
| [95]Díaz(2015) | 33y | G2 | 17wk | NA | NA | Urinary retention | US | Repositioning | Infant viable |
| [96]Ozyurek et al(2015) | 28y | G3P2 | 18^+1^wk | NA | 2 previous caesarean sections with transverse incisions | Ruptured membranes, and recently complaining of pain and pelvic discomfort | US & CT | Considering pre(non-)viable gestation and anomalous placental insertion, perform laparotomy, repositioning during surgery and hysterectomy | Infant nonviable |
| Present case (2015) | 25y | G1P0 | 16^+1^wk | IVF-ET | History of lymphatic tuberculosis at 17y, leading to probable pelvic adhesion and bilateral tubal blockage | Bloody vaginal discharge at 15^+2^wk and increasing lower abdominal pain and urinary retention from 16th week | Pelvic examination & US | Catheter；Repositioning；vaginal delivery at 38 wk. | Infant viable |
| [97]Hassanin et al(2016) | 35y | NA | 15wk | NA | Multiparous | Large painful mass prolapsed  outside the anus | US | Repositioning under general anesthesia; inevitable miscarriage one week later | Infant nonviable |
| [98]Sadath et al(2016) | 23y | G1P0 | 15wk | NA | The septate uterus | Severe back pain as the uterus enlarged | US & MRI | Repositioning; vaginal delivery at 38wk | Infant viable |
| [99]Takami et al(2016) | 34y | G1P0 | 28^+4^wk | NA | Multiple uterine fibroids | Abdominal pain, genital bleeding | MRI | Spontaneous repositioning; CS at 32 wk due to placenta previa | Infant viable, 1570g |
| [100]Kim et al(2016) | 29y | G1P0 | 15^+5^wk | NA | Subserosal myoma | Constipation and low abdominal discomfort | US & MRI | Myomectomy with general anesthesia; spontaneous repositioning; vaginal delivery at 39wk | Infant viable, 3430g |
| [101]Pabuçcu et al(2016) | 35y | G2P2 | 16wk | NA | Previous laparoscopic unilateral (left sided – 5 cm in size) endometrioma excision (endometriosis stage III) before gestations | A total of three admissions for recurrent acute urinary retention | US & MRI | Manual repositioning under anaesthesia failed, and laparotomic ventro-fixation succeeded; spontaneous vaginal delivery at 39wk | Infant viable |
| [102]Schwope et al(2016) | 25y | G8P4 | NA | NA | None | Dysuria; intermittent 2/10 sharp pain in the right lower pelvis that radiated to back | MRI | Manual repositioning under anesthesia with support pessary | NA |

GA=gestational age (weeks); G=gravid; P= partus; IVF-ET= in vitro fertilization and embryo transfer; ICSI=intracytoplasmic sperm injection; US=ultrasound scan; MRI= magnetic resonance imaging; NA= not available; CS= caesarean section; LEEP= loop electrosurgical excision procedure; CT= computed tomography
