# Supplementary material for: Incarceration of the gravid uterus: a case report and literature review
Source: BMC Pregnancy Childbirth. 2019 Nov 8;19:408. doi: 10.1186/s12884-019-2549-3 (PMC6839127; doi:10.1186/s12884-019-2549-3)
Supplement: Supplementary file 4 — Additional file 4. Maternal characteristics with incarceration of the gravid uterus. Based on the 162 case reports, we conducted statistical analysis and summarized the maternal characteristics in the table. (DOCX 19 kb) [file 12884_2019_2549_MOESM4_ESM.docx]

**Additional file 4 Maternal characteristics with incarceration of the gravid uterus**

| Variables | Maternal characteristics based on the available case reports |
| --- | --- |
| Proposed possible risk factors | Previous abdominal surgery history, 24 cases[1,19,20,24,28,38,41,48,51,52,57,58,62,64,66,68,83,91,92,94,96,101]  Abnormal uterus, 23 cases  Unicornuate uterus, 1case[22]  Bicornuate uterus, 11cases[5,6,17,28,38,39,42,59,62]  Didelphic uterus, 8 cases[18,62,69,81,84,88]  Uterus subseptus, 2 cases[32,98]  A heart-shaped uterus with a transverse septum, 1case[41]  Uterine fibroids, 20 cases[2,17,29,36,44,49,54,61,62,72,75,76,86-89,99,100]  Pelvic adhesions, 8 cases[5,38,52,58,64,66,85]  Endometriosis, 5 cases[1,63,64,66,82]  Deep sacral concavity, 2 cases[77,82]  Flat pelvis, 2 cases[9,71]  A history of pelvic inflammatory diseases, 1case[50]  Retroverted uterus, 2 cases[67,74]  Uterine prolapse, 1 case[68] |
| Symptoms | Asymptomatic, 14 cases[23,28,34,37,38,40,41,58,64,81,84,85,89]  Urinary problems, 87 cases  Urinary retention, 66 case [2,9-12,14,15,19,21,24,26,27,29,35,37,42,43,46,54,56,59,60,62,63,65,66,72,75,76,82,83,88,90,92-95,101]  Urinary frequency, 11cases[19,20,26,30,36,37,44,56,70,73,91]  Dysuria, 16cases[16,19,20,24,36,37,45,46,56,57,62,63,68,82,91,102]  Urgency, 5 cases[1,26,27,48,82]  Paradoxical incontinence, 1case [19]  Abdominal pain, 58 cases[19,20,22,23,25-27,30-32,36-38,42,43,45,49-54,56,57,61-63,67,69,70,73,74,87,88,90-92,96,99,100]  Constipation, 11 cases[16,19,26,43,62,67,68,91,92,100]  Vaginal bleeding, 10 cases[16,19,30,51,62,79,99]  Pelvic pain, 11 cases[1,43,44,54,89,96,102]  Back pain, 8 cases[38,44,56,70,73,77,98,102]  Tenesmus, 3 cases[19,30,38]  Perineal pain, 1 case[48]  Large painful mass prolapsed outside the anus, 1 case[97] |
| Diagnosis | Based on symptoms alone, 9 cases[19]  Pelvic examination, 83 cases[1,3,4,6-18,21,22,24-28,30,31,33,35,37,38,43,45,46,48-51,53,54,56,59,60,64-66,68,70,72,74,75,77,82,83,85,86,90,91]  Being confirmed by ultrasound scanning, 59 cases[2,20,23,27,29,31,36-38,41,44,45,48,56,63,65-68,75-77,79,81,84-86,88-92,94-98,100,101]  Magnetic resonance imaging, 42 cases[2,38,51,53,57,62,64,66,67,69,70,74,75,81-84,86-90,93,98-102]  Being observed during surgery, 14 cases[5,20,32,34,39,40,42,47,58,61,71,73,78,80]  Being confirmed by CT, a case[96] |
| Treatments | Manual reposition, 61 cases[1,10,11,14,16,19,21,23,26-28,30,31,35-37,43,45,49,50,54,56,60,62,63,65,66,68,83,85,91-93,95,97,98,102]  Reposition by laparotomy, 11cases[1,10,11,14,16,19,21,23,26-28,30,31,35-37,43,45,49,50,54,56,60,62,63,65,66,68,83,85,91-93,95,97,98,102]  Reposition via laparoscopy, 2 cases[44]  Reposition by a colonoscopy-assisted manipulation, 9 cases[55,82,90]  Spontaneous reposition, 16 cases[2,15,37,41,46,48,62,76,88,89,91,99,100] |
